# Supplementary material for: Influence of droplet coverage on the electrochemical response of planar microelectrodes and potential solving strategies based on nesting concept
Source: PeerJ. 2016 Aug 31;4:e2400. doi: 10.7717/peerj.2400 (PMC5012334; doi:10.7717/peerj.2400)
Supplement: Figure S1 [file peerj-04-2400-s001.docx]

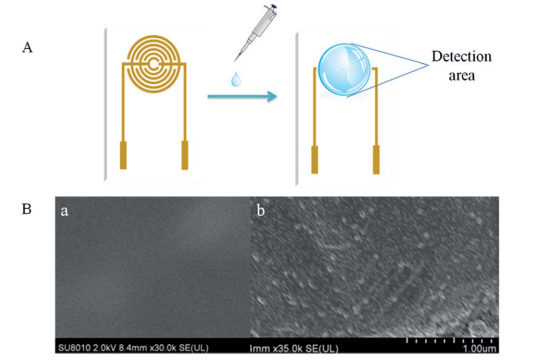


Fig. S1 (A) The SPIM and its detection area, onto which the solution was added for incubation. (B) SEM images. (a) The surface of the bare SPIM and (b) the surface of the SPIM after protein incubation.

Fig S1 was cited from our previous article to characterized the surface of the bare SPIM (a) and the SPIM after protein incubation (b) using scanning electron microscopy (SEM, Fig. 1B). More information: DOI: [10.1039/C5AY02643A](http://dx.doi.org/10.1039/C5AY02643A).

In addition, in order to easy to understand the structure of the SPIM, one figure from a Chinese Patent was added to show the detail.

More information, please refer to the patent: [CN](http://211.157.104.87:8080/sipo/zljs/hyjs-yx-new.jsp?recid=CN201210157775.0&leixin=fmzl&title=%E4%B8%80%E7%A7%8D%E7%94%A8%E4%BA%8E%E5%BE%AE%E7%94%9F%E7%89%A9%E5%BF%AB%E9%80%9F%E6%A3%80%E6%B5%8B%E7%9A%84%E7%94%9F%E7%89%A9%E4%BC%A0%E6%84%9F%E5%99%A8&ipc=G01N27/02(2006.01)I) 202649153U.


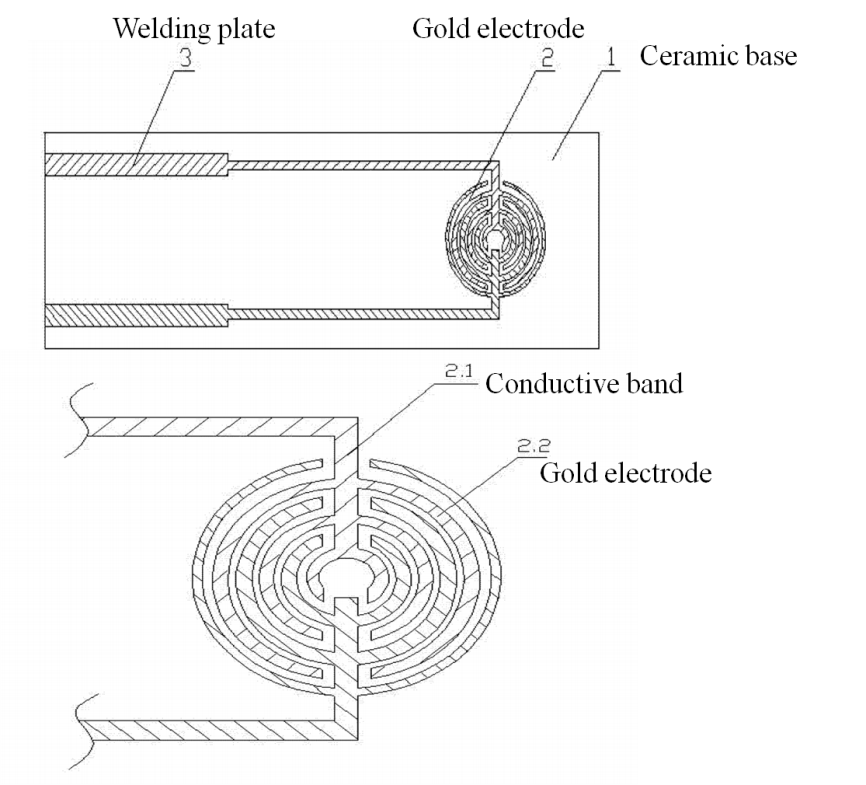


A figure cited from patent ([CN](http://211.157.104.87:8080/sipo/zljs/hyjs-yx-new.jsp?recid=CN201210157775.0&leixin=fmzl&title=%E4%B8%80%E7%A7%8D%E7%94%A8%E4%BA%8E%E5%BE%AE%E7%94%9F%E7%89%A9%E5%BF%AB%E9%80%9F%E6%A3%80%E6%B5%8B%E7%9A%84%E7%94%9F%E7%89%A9%E4%BC%A0%E6%84%9F%E5%99%A8&ipc=G01N27/02(2006.01)I) 202649153U)
