# Supplementary material for: Influence of droplet coverage on the electrochemical response of planar microelectrodes and potential solving strategies based on nesting concept
Source: PeerJ. 2016 Aug 31;4:e2400. doi: 10.7717/peerj.2400 (PMC5012334; doi:10.7717/peerj.2400)
Supplement: Figure S2 [file peerj-04-2400-s002.docx]

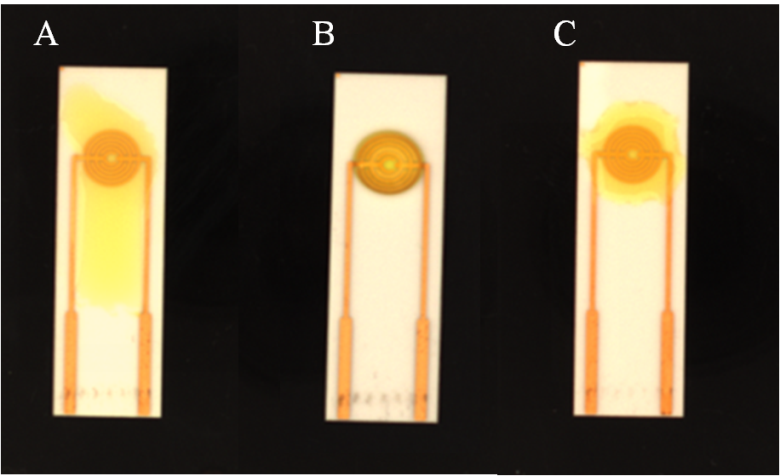


Figure S2. Droplets coverage of the different SPIMs. (A) bare clean SPIM, (B) modified by protein and C) incubation for 2h after the protein immobilization. Yellow solution (*K_3_Fe(CN)_6_/K_4_Fe(CN)_6_*) was used to show the different coverage.
